# FP003B suppresses microcarrier aggregation and enhances cell yield in microcarrier-based mesenchymal stromal cell culture

**DOI:** 10.3389/fbioe.2026.1881013

**Published:** 2026-09-03

**Authors:** Katsuhiko Kida, Daisuke Hatanaka, Shiho Anno, Miki Nakajima, Masaki Kobayashi

**Affiliations:** 1 Biological Research Laboratories, Nissan Chemical Corporation, Shiraoka, Saitama, Japan; 2 Drug Metabolism and Toxicology, Faculty of Pharmaceutical Sciences, Kanazawa University, Kanazawa, Japan; 3 Chemical Research Laboratories, Nissan Chemical Corporation, Funabashi, Chiba, Japan

**Keywords:** aggregation suppression, FCeM, FP003B, mesenchymal stromal cells, microcarrier

## Abstract

**Background:**

Allogeneic transplantation of mesenchymal stromal cells (MSCs) is a promising therapeutic strategy for various diseases. Large-scale expansion is required for allogeneic applications; however, conventional two-dimensional culture systems have limited scalability. Therefore, three-dimensional cultures using a microcarrier (MC) have become increasingly important. A major challenge in MC-based MSC cultures is excessive MC/MSC aggregation, which can compromise cell quality and yields. Here, we present a new approach using functional polymer 003B (FP003B) to suppress excessive aggregation during MC-based MSC cultures.

**Methods:**

Adipose-derived MSCs (ADMSCs) were cultured on multiple MC types using various vessel formats, including 125 mL and 500 mL spinner flasks and a 30 mL disposable bioreactor. FP003B was added either during medium changes or by direct supplementation with concentrated FP003B in D-PBS (+). Aggregation size, cell yield, metabolite profiles, surface marker expression, trilineage differentiation, and paracrine factors were evaluated. Mechanistic studies included particle image velocimetry (PIV) to assess FP003B distribution and confocal microscopy using FITC-labeled FP003B to examine its localization on MC/MSC surfaces. Collagen type I deposition was quantified by enzyme-linked immunosorbent assay.

**Results:**

FP003B significantly reduced the average diameter and area of MC/MSC aggregates and increased the number of harvested viable ADMSCs across multiple MC types and vessel formats. The expression of positive (CD73, CD90, and CD105) and negative (CD11b, CD34, and CD45) surface markers, trilineage differentiation and secretion of prostaglandin E2 and vascular endothelial growth factor from ADMSCs were maintained. In bead-to-bead transfer, FP003B significantly enhanced the expansion ratio and increased cell yields. Mechanistic analyses demonstrated that FP003B localized to the surface of MCs and MSCs, and under agitation, it remained dispersed in the culture medium. Furthermore, collagen type I deposition was significantly reduced in the presence of FP003B, suggesting that suppression of excessive aggregation may contribute to improved cell recovery.

**Conclusion:**

FP003B is a versatile and scalable additive that stabilizes MC-based MSC manufacturing processes. These findings support FP003B as a promising tool for robust and efficient pre-clinical proof-of-concept MSC production.

## 1 Introduction

Mesenchymal stromal cells (MSCs) have attracted considerable attention as a promising cell source owing to their immunomodulatory properties and angiogenic potential, which confer therapeutic benefits across a wide range of diseases (Kanelidis et al., 2017; Wang et al., 2016). Among various transplantation strategies, allogeneic MSC transplantation offers practical advantages, including immediate availability and standardized product quality, making it a feasible option for clinical applications (Aldrich et al., 2021). To meet the demand for allogeneic therapies, large-scale MSC production is essential. However, conventional two-dimensional culture systems are limited in scalability and efficiency, leading to the adoption of three-dimensional culture platforms using microcarriers (MCs) and other technologies (Koh et al., 2020; Kida et al., 2022). MC cultures are particularly suitable for anchorage-dependent cells because they provide an increased surface area for cell attachment and proliferation (Rodrigues et al., 2014). In addition, bead-to-bead transfer, in which fresh MCs are introduced to cultures containing cell-adhered MCs, enables scalable expansion from small- to large-scale systems (Chen et al., 2021). Consequently, the MC-based culture has been widely applied to Madin-Darby canine kidney and Vero cells for vaccine production, as well as to MSCs and induced pluripotent stem cells (iPSCs) for regenerative medicine applications (Koh et al., 2020; Wu et al., 2021; Yang et al., 2019; Schneider et al., 2025). Efficient MSC expansion in MC systems requires optimization of multiple parameters, including the bioreactor configuration, MC type, cell-to-MC ratio, agitation speed, aggregate size, and nutrients and oxygen supply (Lembong et al., 2020; Rafiq et al., 2016). Among these factors, excessive MC/cell aggregation is particularly problematic because it can cause nutrient and oxygen limitations, reduced cell quality, increased heterogeneity, and decreased cell yields (Zhang et al., 2023; Jossen et al., 2016; Ferrari et al., 2012). Because MSCs tend to aggregate during proliferation in MC cultures (Lembong et al., 2020), maintaining controlled aggregation is essential. One strategy to mitigate aggregation is controled by specific bioreactor designs, impeller configurations, or MC properties, which limit their general applicability (Lembong et al., 2020; Rafiq et al., 2016; Ferrari et al., 2012). Another strategy is the use of chemical anti-aggregation additives, such as the surfactant Pluronic F-68 and viscosity-modifying polymers like methylcellulose (Negrete et al., 2008; Otsuji et al., 2014). However, there are limited reports demonstrating effective aggregation control using these additives in MC cultures of MSCs.

FP003B is a microbial exopolysaccharide analogue of gellan gum that is similar to FP001 and FP003. Media supplemented with FP001, FP003, or FP003B allow single cells, spheroids, and organoids to remain suspended without agitation. These polymers have enabled suspension culture of iPSCs, HepaRG cells, human vascular smooth muscle cells, and iPSC-derived intestinal organoids (Otsuji et al., 2014; Horiguchi et al., 2021; Higuchi et al., 2016; Uchida et al., 2023; Ogawa et al., 2022). Furthermore, FP003 has been reported to suppress iPSC spheroid aggregation under low-agitation conditions (Horiguchi et al., 2021). Compared with FP003, FP003B was developed by optimizing polymer molecular weight to reduce the size of microgels formed in culture medium. This results in a greater number of dispersed microgels per unit mass, which may improve dispersion characteristics and make FP003B particularly suitable for regulating interactions among MCs and cells during agitation culture. However, whether FP003B can prevent excessive aggregation in MC-based MSC cultures is unclear.

In this study, we investigated whether FP003B could serve as a scalable and vessel-independent approach to control MC/MSC aggregation in adipose-derived MSC (ADMSC) cultures. We systematically evaluated its effects on aggregate size, expansion efficiency, and process robustness across multiple MC types and bioreactor formats, including bead-to-bead transfer. Our findings provide a practical approach to stabilizing MC-based MSC manufacturing processes for large-scale applications.

## 2 Materials and methods

### 2.1 Chemicals and reagents

Human ADMSCs (Male, 21 years old, Np = 2) were from CellSource Co., Ltd. (Tokyo, Japan). StemFit For Mesenchymal Stem Cell was purchased from Ajinomoto Healthy Supply Co., Inc. (Tokyo, Japan). Corning low-concentration Synthemax II microcarriers (Synthemax II MC), Corning 125 mL and 500 mL disposable spinner flasks, and 24-well cell culture plate were obtained from Corning Incorporated (NY, United States). SCAPOVA CL PVA microcarriers with collagen coating (SCAPOVA MC) were purchased from KURARAY Co., Ltd (Tokyo, Japan). The ABLE 30 mL Disposable Bioreactor was from ABLE Corporation and Biott Co., Ltd. (Tokyo, Japan). The FP003B contained in FCeM Advance-CR Preparation Kit (commercial product name, hereafter referred to as FCeM) and FITC-labeled FP003B were from Nissan Chemical Corporation (Tokyo, Japan). Dulbecco’s Phosphate-Buffered Saline (D-PBS) (−) and low-glucose DMEM were obtained from FUJIFILM Wako Pure Chemical Corporation (Osaka, Japan). D-PBS with calcium and magnesium (D-PBS [+]), fetal bovine serum (FBS), TrypLE Select Enzyme (1×, no phenol red), and propidium iodide were from Thermo Fisher Scientific (MA, United States). Via1-Cassettes, Reagent A100, and Reagent B were purchased from ChemoMetec (Allerod, Denmark). PluriStrainer (40 µm) was from pluriSelect (Leipzig, Germany). Anti-CD11b-FITC (clone no. M1/70) and VEGF enzyme-linked immunosorbent assay (ELISA) kit were from Abcam (MA, United States). Anti-CD34-PE (clone no. 8G12), anti-CD45-Alexa Fluor 700 (clone no. HI30), anti-CD73-BV421 (clone no. AD2), anti-CD90-APC (clone no. 5E10), and anti-CD105-BV510 (clone no. 266) were obtained from BD Biosciences (CA, United States). iMatrix-511 silk was obtained from Nippi incorporated (Tokyo, Japan). Adipogenic differentiation medium, Osteogenic differentiation medium, and Chondrogenic differentiation medium were obtained from PromoCell (Heidelberg, Germany). Oil-Red O solution, Alizarin Red S solution, and Alcian Blue solution were purchased from Bio Future Technologies (Tokyo, Japan). 96-well U-bottom plate was obtained from SUMITOMO BAKELITE CO., LTD. (Tokyo, Japan). The PGE_2_ ELISA kit was obtained from Enzo Life Sciences (NY, United States). Methylcellulose was obtained from Shin-Etsu Chemical Co., Ltd (Tokyo, Japan). The Cell Explorer Live Cell Labeling Kit *Blue Fluorescence was purchased from AAT Bioquest (CA, United States). Tissue culture plates (1 Well) were obtained from CELLTREAT Scientific Products (MA, United States). Pepsin (from porcine gastric mucosa) was from Merck (NJ, United States). The human collagen type I ELISA kit was obtained from ACEL Inc (Sagamihara, Japan).

### 2.2 Culture of ADMSCs using a 125 mL spinner flask and MCs

All experiments were conducted using ADMSCs derived from a single donor and independent biological replicates refer to separate culture experiments performed under identical conditions using the same donor source. ADMSCs (2 × 10^4^ cells/ml) were seeded into 80 mL of culture medium containing either 1.11 g of Synthemax II MC (total surface area: 399 cm^2^) or 153 mg of SCAPOVA MC (total surface area: 398 cm^2^) and then transferred into a 125 mL disposable spinner flask. The initial seeding density was 4,000 cells/cm^2^ for both MC types. The cells and MCs were intermittently stirred (for 1 min every 30 min) at 50 rpm for 10 cycles, followed by continuous agitation at 50 rpm. The cultures were maintained at 37 °C in a humidified atmosphere of 5% CO_2_ and 95% air. After 24 h, FP003B was added using one of the following methods. For medium exchanges, 53 mL of culture medium was removed and replaced with an equal volume of fresh medium with or without 0.0075% FP003B (final concentration: 0.005%). Alternatively, 4 mL of D-PBS (+) with or without 0.1% FP003B (final concentration: 0.005%) was directly added to the culture medium. Agitation was maintained at 50 rpm throughout the culture period. After 96 h, cells were harvested using TrypLE Select Enzyme (1×, no phenol red). Detached cells and MCs were separated using a 40 µm pluriStrainer. Harvested cells were counted using a TC20 Automated Cell Counter (Bio-Rad Laboratories; CA, United States). Residual cells remaining on MCs were quantified using a NucleoCounter NC-200 CellCounter (ChemoMetec) with Via1-Cassettes, Reagent A100, and Reagent B according to the manufacturer’s instructions. Aggregate size was quantified using image-based analysis. Different analysis pipelines were used depending on the MC type due to differences in image contrast. For Synthemax II MC, aggregate size was determined using the automated Cell3iMager duos system (SCREEN Holdings Co. Ltd.; Kyoto, Japan). For SCAPOVA MC, images were analyzed using ImageJ software to enable accurate segmentation under these conditions, and aggregate diameters were subsequently measured. For each condition, aggregates were imaged from six fields of view. At least 10 aggregates were analyzed per sample in each biological replicate. Glucose, lactate, and ammonium ion (NH_4_^+^) concentrations in the culture medium were analyzed using a BioProfile FLEX2 (Nova Biomedical; MA, United States).

### 2.3 Culture of ADMSCs using a 500 mL spinner flask and synthemax II MC

ADMSCs (2 × 10^4^ cells/ml) were seeded into 300 mL of culture medium containing 4.17 g of Synthemax II MC and transferred into a 500 mL disposable spinner flask. The cells and MCs were intermittently stirred (for 1 min every 30 min) at 50 rpm for 10 cycles, followed by continuous agitation at 50 rpm. The culture was maintained at 37 °C in a humidified atmosphere of 5% CO_2_ and 95% air. After 24 h, FP003B was added using the following method. From the culture medium, 200 mL was removed and replaced with an equal volume of fresh medium with or without 0.0075% FP003B (final concentration: 0.005%). Agitation was maintained at 50 rpm throughout the culture period. After 96 h, cells were harvested using TrypLE Select Enzyme (1×, no phenol red). Detached cells and MCs were separated using a 40 µm pluriStrainer. Harvested cells and residual cells on MCs were measured as above.

### 2.4 Bead-to-bead transfer of ADMSCs using a 125 mL spinner flask and synthemax II MC

ADMSCs (2 × 10^4^ cells/ml) were seeded into 80 mL of culture medium containing 1.11 g of Synthemax II MC and transferred into a 125 mL disposable spinner flask. The cells and MCs were intermittently stirred (for 1 min every 30 min) at 50 rpm for 10 cycles, followed by continuous agitation at 50 rpm. The culture was maintained at 37 °C in a humidified atmosphere of 5% CO_2_ and 95% air. After 24 h, FP003B was added using the following method. From the culture medium, 53 mL was removed and replaced with an equal volume of fresh medium with or without 0.0075% FP003B (final concentration: 0.005%). Agitation was maintained at 50 rpm throughout the culture period. After 120 h from initial seeding, cells were harvested using TrypLE Select Enzyme (1×, no phenol red). Detached cells and MCs were separated using a 40 µm pluriStrainer. Harvested cells and residual cells on MCs were measured as above. Then MC/MSC aggregates (2 × 10^6^ cells) were reseeded into 80 mL of a culture medium containing 0.85 g of Synthemax II MC and transferred into a 125 mL disposable spinner flask. The cells and MCs were intermittently stirred (for 1 min every 30 min) at 50 rpm for 10 cycles, followed by continuous agitation at 50 rpm. After 24 h from bead-to-bead transfer, FP003B was added using the following method. For medium exchanges, 53 mL of culture medium was removed and replaced with an equal volume of fresh medium with or without 0.0075% FP003B (final concentration: 0.005%). Agitation was maintained at 50 rpm throughout the culture period. After 264 h from the initial seeding (120 h in the first culture and an additional 144 h after bead-to-bead transfer), cells were harvested using TrypLE Select Enzyme (1×, no phenol red). Detached cells and MCs were separated using a 40 µm pluriStrainer. Harvested cells and residual cells on MCs were measured as above.

### 2.5 Culture of ADMSCs using a 30 mL bioreactor and synthemax II MC

ADMSCs (2 × 10^4^ cells/ml) were seeded into 30 mL of culture medium containing 0.416 g of Synthemax II MC and then dispensed into an ABLE 30 mL disposable bioreactor. The cells and MCs were intermittently stirred (for 1 min every 30 min) at 50 rpm for 10 cycles, followed by continuous agitation at 50 rpm. The culture was maintained at 37 °C in a humidified atmosphere of 5% CO_2_ and 95% air. After 24 h, FP003B was added using the following method. A total of 1.5 mL of D-PBS (+) with or without 0.1% FP003B (final concentration: 0.005%) was directly added to the culture medium. Agitation was maintained at 50 rpm throughout the culture period. After 96 h, cells were harvested using TrypLE Select Enzyme (1×, no phenol red). Detached cells and MCs were separated using a 40 µm pluriStrainer. Harvested cells and residual cells on MCs were measured as above.

### 2.6 Flow cytometry analysis

ADMSCs were incubated with CD73, CD90, CD105, CD11b, CD34, and CD45 antibodies following the antibody reagent manufacturer’s instructions. The ADMSCs were centrifuged at 300 g for 3 min and washed with wash buffer (2% FBS in D-PBS [-]). The cells and antibodies were incubated at 25 °C for 30 min in the dark. After the ADMSCs were washed twice with wash buffer, propidium iodide was added to stain dead cells. Antibody-labeled cells were measured using a BD LSRFortessa X-20 (BD Biosciences), and flow cytometry data were analyzed using FlowJo v10.6.1 software (BD Biosciences).

### 2.7 Trilineage mesenchymal differentiation

After obtaining single-cell suspensions at t = 120 h, cells were seeded for each differentiation. Adipogenic differentiation was performed using Adipogenic differentiation medium. Briefly, cells were seeded with growth medium in 24-well plates coated with iMatrix-511 silk (0.5 μg/cm^2^) and cultured until 90% confluence. The medium was then replaced with differentiation medium and cultured for 11 days with medium changes every third day. Cells were stained with Oil-Red O solution according to the manufacturer’s instructions. Osteogenic differentiation was conducted using Osteogenic differentiation medium. Cells were seeded 24-well plates coated with iMatrix-511 silk (0.5 μg/cm^2^), cultured until 100% confluence, and then treated with differentiation medium for 12 days with medium changes every third day. Cells were stained with Alizarin Red S solution according to the manufacturer’s instructions. Chondrogenic differentiation was performed using Chondrogenic differentiation medium. Briefly, 2 × 10^5^ cells were seeded in low-glucose DMEM containing 10% (v/v) FBS in 96-well U-bottom plates. After 2 days of culture, the medium was replaced with differentiation medium and cultured for 19 days with medium changes every third day. Cells were stained with Alcian Blue solution. Images of stained cells were acquired using an inverted microscope (IX73) (Olympus Corporation; Tokyo, Japan).

### 2.8 Measurement of PGE_2_ and VEGF by ELISA

ADMSCs were seeded at a density of 8 × 10^4^ cells/mL in growth medium and dispensed into an iMatrix-511 silk-coated 24-well plate at 1 mL/well. These plates were cultured unstirred for 48 h, after which the cells were switched to fresh growth medium. Cells were cultured for a further 24 h, after which the culture supernatants were harvested. The quantification of PGE_2_ and VEGF in culture supernatants was performed in accordance with the manufacturer’s protocols.

### 2.9 Confirmation of the presence of FP003B in the medium during agitation culture

Structures of FP003B in the culture medium were visualized by irradiating FP003B and the medium, which contained MCs, with a green laser sheet formed by a PIV Laser G2000 (KATO KOKEN; Isehara, Japan). The wavelength and laser intensity were 532 nm and 3.2 A, respectively. The motion of FP003B and the medium were recorded using a Z50 (NIKON CORPORATION; Tokyo, Japan).

### 2.10 Fluorescence localization of FP003B on MC/MSC aggregate surfaces

FITC-labeled FP003B was used for the culture. Live cells were stained using the Cell Explorer Live Cell Labeling Kit *Blue Fluorescence according to the standard protocol. They were imaged using a FLUOVIEW FV1000 (Olympus Corporation) at 405 and 488 nm for live cells and FITC-labeled FP003B, respectively. Sixteen images were obtained every 20 µm in the Z-axis direction.

### 2.11 Measurement of collagen type I deposition by ELISA

ADMSCs were cultured in a 125 mL flask and Synthemax II MC for 120 h as above. Cultured MC/MSC aggregates were transferred into four tissue culture plates (1well), and the number of MC/MSC aggregates was counted using a Cell3iMager duos. Sample preparation for human collagen type I ELISA followed the manufacturer’s instructions. All MC/MSC aggregates were collected and washed with D-PBS (−) and immersed in 0.1 mg/ml pepsin dissolved in acetic acid at 4 °C for 24 h to make atelo-collagen from collagen type I. After 24 h, supernatants were collected, and one-third the volume of neutralization solution containing 200 mM Tris and 150 mM NaCl was added and mixed well. Samples were measured by a human collagen type I ELISA kit in accordance with the manufacturer’s protocols.

### 2.12 Measurement of viscosity

The viscosity of each culture medium was determined using an E-type rotational viscometer equipped with a cone-plate geometry (TV-22L) (Toki Sangyo; Tokyo, Japan). A standard cone–plate rotor (1° 34′× R24) was used. Measurements were conducted at 25 °C using the M range (full-scale torque: 67.37 μN·m) at a fixed rotation speed of 100 rpm.

### 2.13 Statistical analysis

**FIGURE 1:**
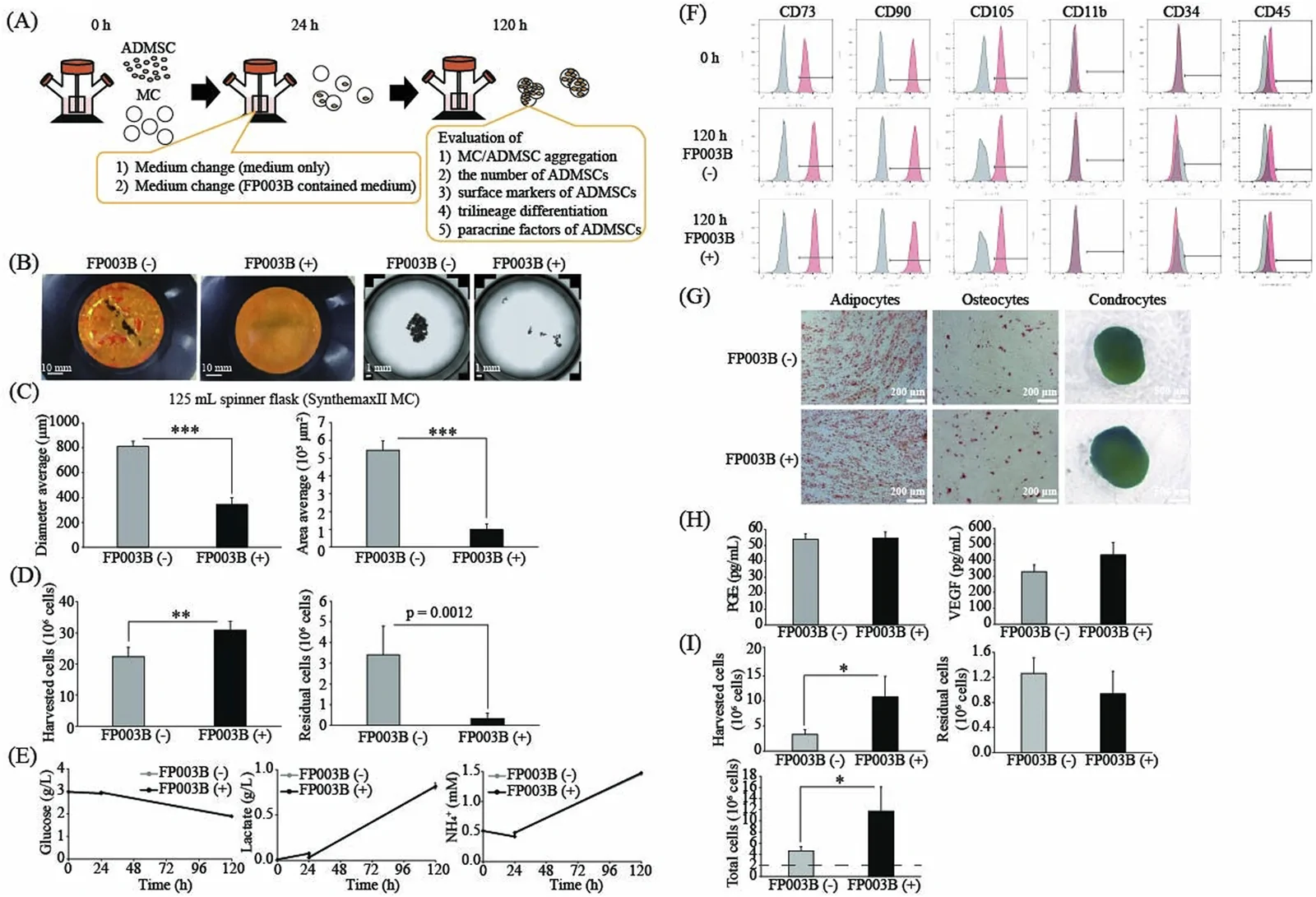
Suppression of MC/MSC aggregation using FP003B in a 125 mL spinner flask. **(A)** Schematic illustration of the experimental design. Orange and white circles show ADMSCs and MCs, respectively. **(B)** Images of the bottom of the spinner flask and MC/MSC aggregates. Scale bars = 10 mm and 1 mm, respectively. **(C)** The average diameter and area of MC/MSC aggregates. ***p < 0.001, compared with FP003B (−). Bars indicate mean ± SD (n = 5). **(D)** The number of harvested and residual ADMSCs. **p < 0.01, compared with FP003B (−). Bars indicate mean ± SD (n = 5). **(E)** The concentrations of glucose, lactate, and NH_4_^+^ in supernatants. Plots indicate mean ± SD (n = 3). **(F)** Representative flow cytometry histograms of human ADMSCs. Gray histograms are isotype controls, and red histograms are experimental samples. **(G)** Trilineage differentiation of ADMSCs into adipogenic, osteogenic, and chondrogenic lineages. Scale bar = 200 μm and 500 μm, respectively. **(H)** The secretion levels of PGE_2_ and VEGF from ADMSCs. Bars indicate mean ± SD (n = 3). **(I)** The number of harvested, residual, and total ADMSCs in bead-to-bead transfers. The dotted line indicates the number of reseeding cells at 120 h. *p < 0.05, compared with FP003B (−). Bars indicate mean ± SD (n = 3).

**FIGURE 2:**
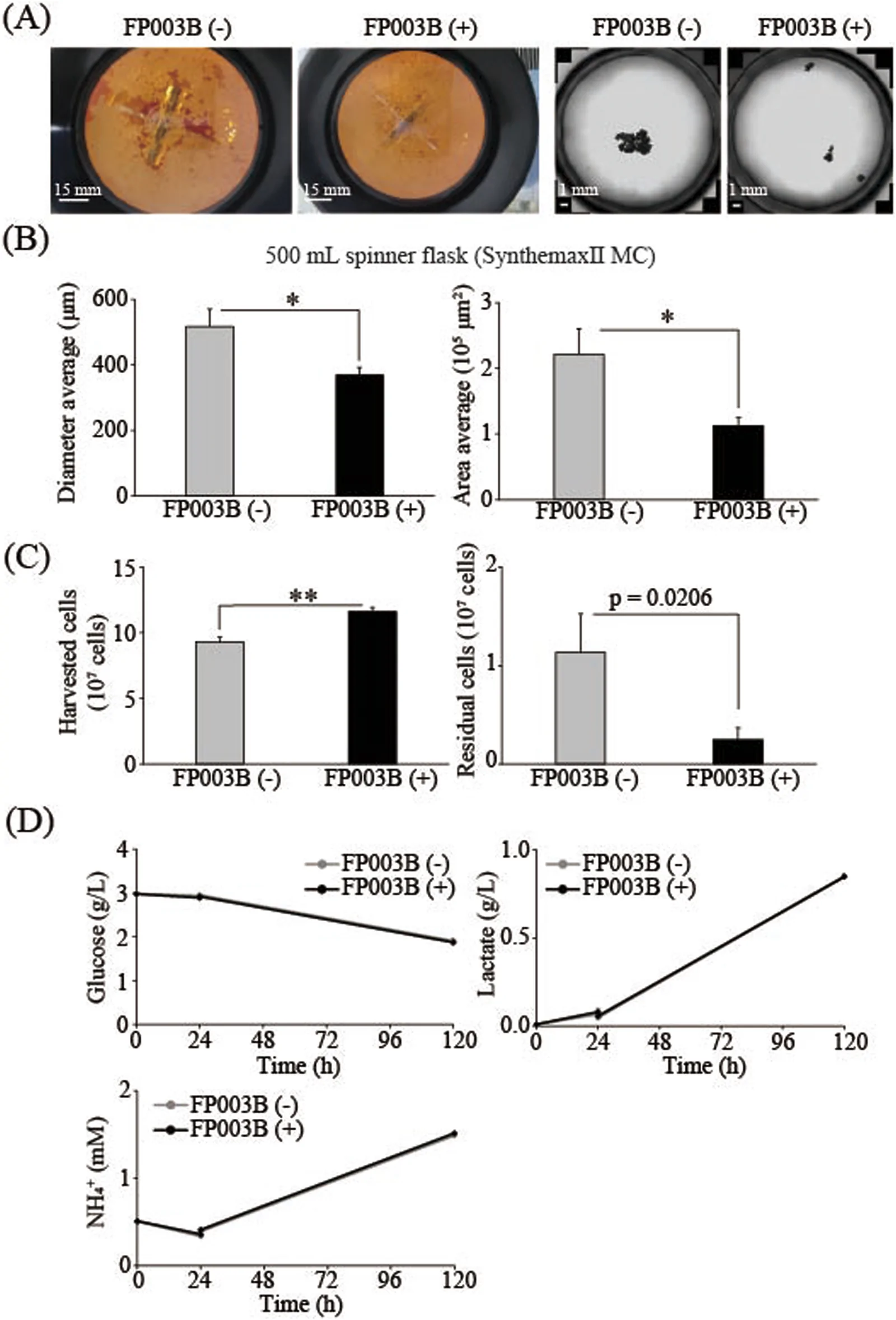
Suppression of MC/MSC aggregation using FP003B in a 500 mL spinner flask. **(A)** Images of the bottom of the spinner flask and MC/MSC aggregates. Scale bars = 15 mm and 1 mm, respectively. **(B)** The average diameter and area of MC/MSC aggregates. *p < 0.05, compared with FP003B (−). Bars indicate mean ± SD (n = 3). **(C)** The number of harvested and residual ADMSCs. *p < 0.05, **p < 0.01, compared with FP003B (−). Bars indicate mean ± SD (n = 3). **(D)** The concentrations of glucose, lactate, and NH_4_^+^ in supernatants. Plots indicate mean ± SD (n = 3).

All results are presented as mean ± standard deviation (SD) from three or five independent biological replicates. Statistical significance was assessed using two-tailed Student’s t-tests for pairwise comparisons. To account for multiple comparisons, p-values were adjusted using the Benjamini-Hochberg false discovery rate correction within each set of related comparisons corresponding to distinct experimental designs (e.g., Figures 1C–D,G, 2, 4). Adjusted p-values are reported in Supplementary Table S1. For experiments involving three-group comparisons (Figure 4), statistical analysis was performed using one-way ANOVA followed by Tukey’s post hoc test for multiple comparisons. Statistical analyses were performed using EXSUS ver. 10.2 (Arm Systex; Osaka, Japan), and a significance level of p < 0.05 was applied. Due to the limited sample size (n = 3–5), assumptions of normality and homogeneity of variance were not formally evaluated. However, parametric tests were retained as exploratory analyses, assuming approximate normality of biological measurements. In addition, effect size estimates derived from such small samples may be unstable and potentially inflated.

## 3 Results

### 3.1 Suppression of MC/MSC aggregation using FP003B in a 125 mL spinner flask

First, we cultured ADMSCs on MCs in a 125 mL spinner flask to confirm whether FP003B can suppress MC/MSC aggregation, which was assessed by visual inspection and using a Cell3iMager duos. A schematic illustration of the experimental design is shown in Figure 1A. After 120 h of culture, the size of MC/MSC aggregates was qualitatively reduced by the addition of FP003B (Figure 1B; Supplementary Videos S1, S2). The average diameter and area of the aggregates were quantified using a Cell3iMager duos. FP003B significantly decreased both parameters (0.42- and 0.18-fold compared with control, respectively, p < 0.001) (Figure 1C). To investigate whether FP003B improves cell recovery by suppressing aggregation, we measured the number of harvested cells and residual cells remaining on MCs. The number of harvested cells significantly increased (1.38-fold, p < 0.01), whereas the number of residual cells significantly decreased (0.1-fold, p = 0.0012) in the presence of FP003B (Figure 1D). We also monitored glucose, lactate, and NH_4_^+^ concentrations in the supernatants as indicators of cell growth, and these showed similar trends between the groups at each time point (Figure 1E). Finally, to examine whether FP003B affects ADMSC characteristics, we analyzed surface marker expression, trilineage mesenchymal differentiation, and secretion of PGE_2_ and VEGF. Positive markers (CD73, CD90, and CD105) and negative markers (CD11b, CD34, and CD45) were maintained regardless of FP003B treatment (Figure 1F; Supplementary Table S2). In addition, ADMSCs retained trilineage mesenchymal differentiation capacity, including adipogenic, osteogenic, and chondrogenic differentiation (Figure 1G), with negative staining confirmed at the initiation of differentiation (Supplementary Figure S1). Furthermore, the secretion levels of PGE_2_ and VEGF were not significantly different in both conditions (Figure 1H). From these results, FP003B effectively suppressed MC/MSC aggregation and improved cell recovery while maintaining MSC-related characteristics examined in this study, as evidenced by preserved surface marker expression, trilineage differentiation capacity, and paracrine factor secretion.

When scaling up MSC culture using MCs, bead-to-bead transfer is generally employed because it eliminates the need for enzymatic dissociation and simplifies the manufacturing process. Therefore, we examined the effects of FP003B on bead-to-bead transfer using ADMSCs. After 264 h of culture, the number of harvested cells significantly increased (3.2-fold, p < 0.05), whereas the number of residual cells did not significantly decrease in the presence of FP003B (0.74-fold) (Figure 1I). In addition, the total cell number was significantly higher in the FP003B-treated group (2.53-fold, p < 0.05) (Figure 1I). These findings indicate that FP003B suppressed excessive MC/MSC aggregation and enhanced the recovery of cells. Furthermore, FP003B improved the expansion ratio of ADMSCs during bead-to-bead transfer by suppressing MC/MSC aggregation.

### 3.2 Suppression of MC/MSC aggregation using FP003B in a 500 mL spinner flask

To determine whether FP003B can also suppress MC/MSC aggregation in a large-scale culture, we performed an MC-based ADMSC culture in a 500 mL spinner flask. After 120 h of culture, FP003B qualitatively reduced the size of MC/MSC aggregates (Figure 2A), and the addition of FP003B significantly decreased the average diameter (0.71-fold, p < 0.05) and area (0.51-fold, p < 0.05) of the aggregates (Figure 2B). Furthermore, the number of harvested cells significantly increased (1.25-fold, p < 0.01), whereas the number of residual cells significantly decreased in the presence of FP003B (0.22-fold, p = 0.0206) (Figure 2C). We also measured glucose, lactate, and NH_4_^+^ concentrations in the supernatants to monitor cell growth, and these values were comparable between groups at all time points (Figure 2D). Taken together, FP003B suppressed excessive MC/MSC aggregation and increased the recovery of ADMSCs in a large-scale culture, consistent with the results observed in a small-scale culture.

### 3.3 Comparison of methods for FP003B supplementation

It has been reported that increasing the number of operational steps can lead to inconsistent cell quality in cell manufacturing processes (Lipsitz et al., 2016). In our process, several steps were required to add a medium containing FP003B to the vessel (Figure 4A, upper panel). If FP003B could be added directly to the culture medium, the number of required steps could be reduced (Figure 4A, lower panel). Therefore, we examined whether directly adding FP003B diluted in D-PBS (+) to the medium would exert the same effect as performing a full medium exchange, thereby reducing the number of steps in the manufacturing workflow. A D-PBS (+)-only condition (Add D-PBS) was included as a vehicle control. The direct addition of FP003B containing D-PBS (+) (Add FP003B) significantly decreased the average diameter (0.39-fold, p < 0.001) and area (0.16-fold, p < 0.001) of the aggregates (Figures 4B,C). On the other hand, the size and area of MC/MSC aggregates were comparable between the medium-change method (Med-chg FP003B) and Add (FP003B) (Figures 4B,C). Furthermore, the numbers of harvested and residual ADMSCs in Add (FP003B) were significantly increased (2.06-fold, p < 0.01) and decreased (0.03-fold, p < 0.01) compared with Add (D-PBS) and they were nearly identical between Med-chg (FP003B) and Add (FP003B) (Figure 4D). Thus, FP003B suppressed excessive MC/MSC aggregation not only when added via medium exchange but also when directly introduced into the culture medium as FP003B-D-PBS (+) solution.

### 3.4 Suppression of MC/MSC aggregation across various types of MCs and culture vessels

**FIGURE 3:**
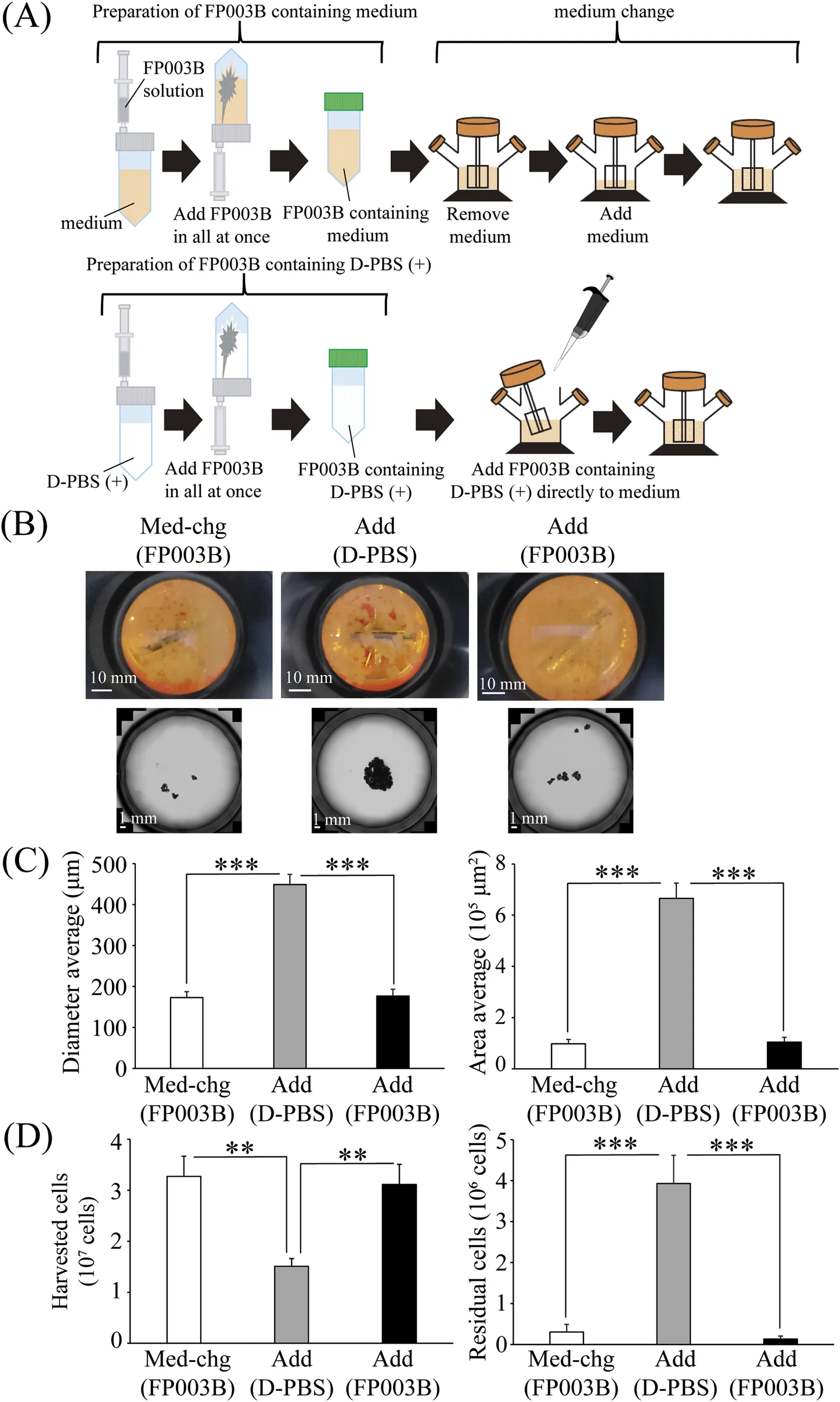
Comparison of methods for FP003B supplementation. **(A)** Schematic illustration of medium change and direct addition methods. The upper panel shows the method of medium change, and the lower panel shows the addition of FP003B containing D-PBS (+). **(B)** Images of the bottom of the spinner flask and MC/MSC aggregates. Med-chg (FP003B), add (D-PBS) and add (FP003B) mean medium change with FP003B, addition of D-PBS (+) only and addition of FP003B containing D-PBS (+), respectively. Scale bars = 10 mm and 1 mm, respectively. **(C)** The average diameter and area of MC/MSC aggregates. ***p < 0.001, compared with add (D-PBS). Bars indicate mean ± SD (n = 3). **(D)** The number of harvested and residual ADMSCs. **p < 0.01, ***p < 0.001, compared with add (D-PBS). Bars indicate mean ± SD (n = 3).

**FIGURE 4:**
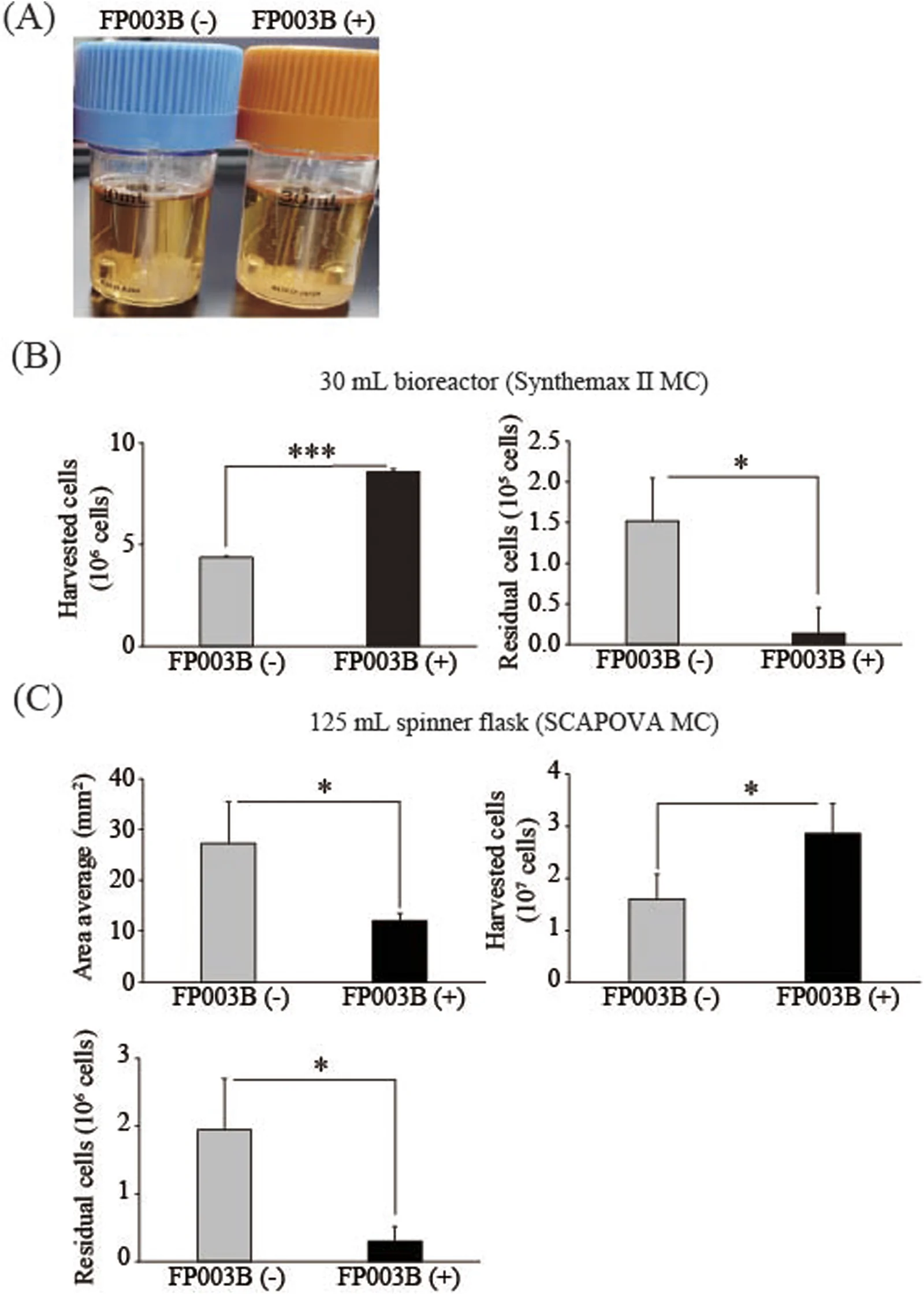
Suppression of MC/MSC aggregation across various types of MCs and culture vessels. **(A)** Images of MC/MSC aggregates in an ABLE 30 mL disposable bioreactor. Left vessel: FP003B (−), Right Vessel: FP003B (+). **(B)** The number of harvested and residual ADMSCs using an ABLE 30 mL disposable bioreactor. *p < 0.05, ***p < 0.001, compared with FP003B (−). Bars indicate mean ± SD (n = 3). **(C)** The average diameter of MC/MSC aggregates and the number of harvested and residual ADMSCs using SCAPOVA MC. *p < 0.05, compared with FP003B (−). Bars indicate mean ± SD (n = 3).

The observation of consistent effects across different types of MCs and culture vessels is highly important in terms of applicability and versatility. Therefore, we investigated the effects of FP003B across various combinations of MC types and culture vessels using ADMSCs. When using Synthemax II MC in an ABLE 30 mL disposable bioreactor, substantial MC/MSC aggregation was observed in the control group compared with the FP003B-treated group (Figure 4A). Due to the large amount of aggregation, only the number of harvested cells and residual cells on the MCs was quantified in this condition. The number of harvested cells significantly increased (1.97-fold, p < 0.001), whereas the number of residual cells significantly decreased following FP003B addition (0.09-fold, p < 0.05) (Figure 3B).

In cultures using SCAPOVA MC in a 125 mL spinner flask, FP003B significantly reduced the average area of MC/MSC aggregates (0.44-fold, p < 0.05) (Figure 3C). Furthermore, FP003B treatment significantly increased the number of harvested cells (1.78-fold, p < 0.05) and significantly decreased the number of residual cells (0.15-fold, p < 0.05) (Figure 3D). Together, these results demonstrate that FP003B suppressed excessive MC/MSC aggregation and improved cell recovery across multiple combinations of MC types and culture vessel formats.

### 3.5 The mechanism underlying the suppression of MC/MSC aggregation and the improved cell recovery

**FIGURE 5:**
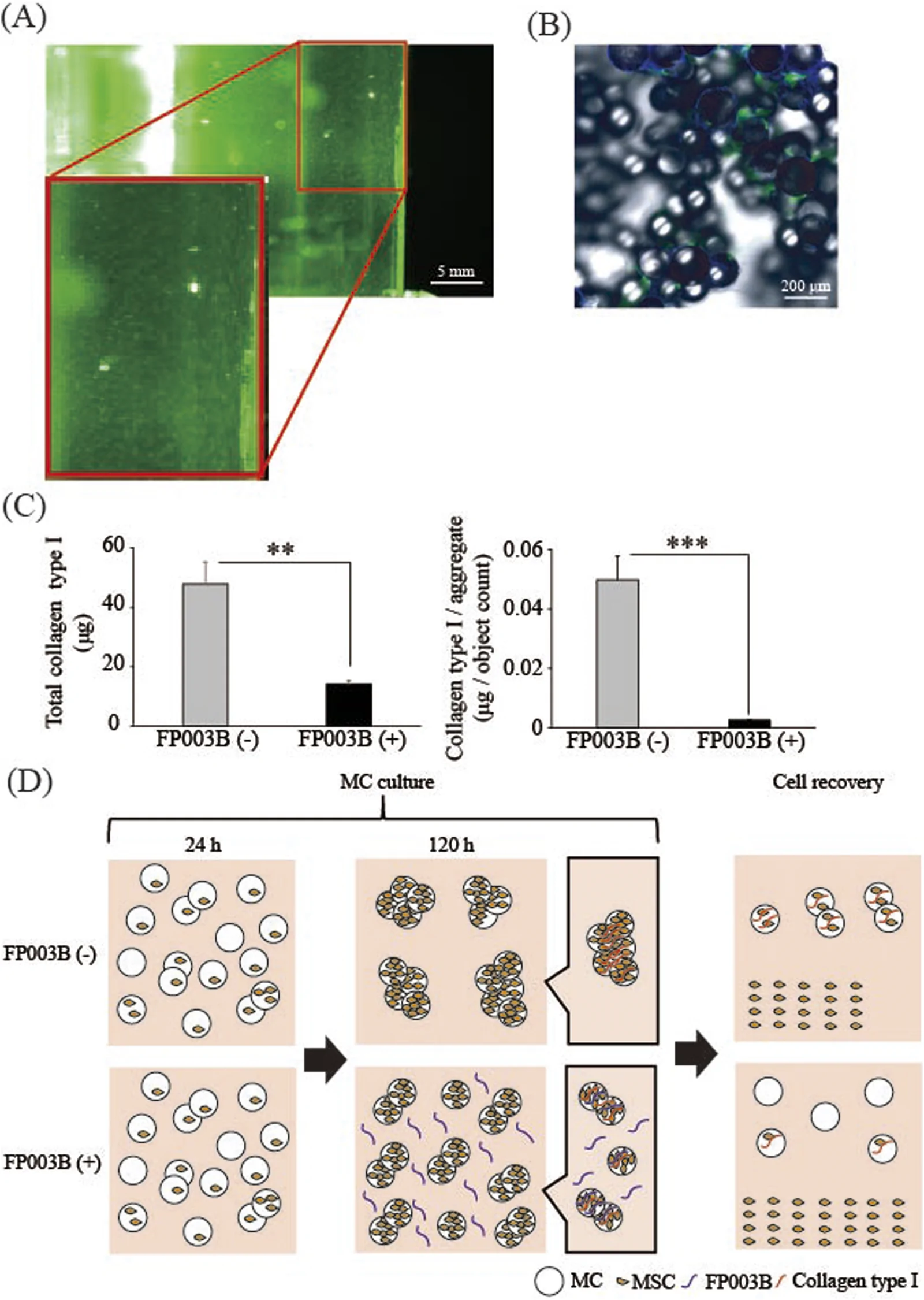
The mechanisms underlying the suppression of MC/MSC aggregation and enhancement of cell recovery. **(A)** The image of FP003B in the culture medium being analyzed by a PIV laser. The red frame indicates the enlarged area. Scale bar = 5 mm. **(B)** The image of FP003B localization on MC and ADMSC surfaces. Green indicates FITC-labeled FP003B, and blue indicates stained live ADMSCs. Scale bar = 200 µm. **(C)** The deposition levels of total collagen type I and the average amount of collagen type I per MC/MSC aggregates. **p < 0.01, ***p < 0.001, compared with FP003B (−). Bars indicate mean ± SD (n = 3). **(D)** Schematic illustration of the mechanisms underlying aggregation suppression and enhancement of cell recovery.

FP003B forms an ion-dependent network structure in the medium. To investigate the mechanism by which FP003B suppresses excessive MC/MSC aggregation, we employed particle image velocimetry (PIV) laser imaging to visualize the behavior of FP003B in the culture medium under both static and agitated conditions. Using PIV laser imaging, FP003B was visualized as small particles in static conditions (Figure 5A), and these particles were also observed in the medium under agitated conditions (Supplementary Video S3). Next, to confirm the presence of FP003B on the surfaces of MCs and ADMSCs, we performed fluorescence imaging using FITC-labeled FP003B. FP003B was clearly detected on the surface of MC/MSC aggregates (Figure 5B, Supplementary Video S4). These results indicate that FP003B not only remained dispersed in the culture medium during agitation but also localized to the surfaces of MC/MSC aggregates.

In the preceding experiments, the firmness of aggregates in the control group appeared higher than in the FP003B-treated group. Based on this observation, we hypothesized that extracellular matrix deposition may differ between the groups and measured collagen type I levels. Both the total amount of collagen type I and collagen type I per aggregate were significantly lower in the FP003B-treated group than in the control group (0.29-fold, p < 0.01 and 0.05-fold, p < 0.001, respectively) (Figure 5C). A schematic representation of the proposed mechanism underlying aggregation suppression and enhanced cell recovery is shown in Figure 5D. Collectively, these findings suggest that FP003B remained present at an appropriate particle size in the culture medium and localized to MC/MSC aggregate surfaces, thereby mitigating excessive aggregation, which may contribute to improved enzymatic detachment during harvesting and enhanced cell recovery.

## 4 Discussion

MCs are adapted to culture various types of anchorage-dependent cells (Yu et al., 2012; Genzel et al., 2006; Zhao et al., 2024), and they are suitable for large-scale cell cultures in bioreactors. However, MC/cell aggregation occurs in MC-based cultures, and it affects nutrient and oxygen supply, cell quality, heterogeneity, and cell yield (Zhang et al., 2023; Jossen et al., 2016; Ferrari et al., 2012). Considering the significance of this, we initiated this study to explore solutions to the problem of MC/MSC aggregation.

There are several advantages to using this material for suppressing MC/MSC aggregation. One advantage is that FP003B is applicable across various culture scales, MC types, and vessel formats. In this study, we evaluated two types of MCs (Synthemax II MC and SCAPOVA MC), two types of culture vessels (a spinner flask and an ABLE disposable bioreactor), and three different working volumes (30, 80, and 300 mL). Despite differences in MC materials, impeller geometry, and culture scales, FP003B consistently suppressed excessive MC/MSC aggregation across all conditions (Figures 1C, 2B, 3A,C). These results indicate that MC/MSC aggregate size can be easily controlled simply by adding FP003B, highlighting its potential to broaden the operational design space. Another important advantage of FP003B in MC-based MSC culture relates to the potential to scale it up. Bead-to-bead transfer is commonly employed for generating large quantities of MSCs in a single batch (Chen et al., 2021). In our experiments, excessive MC/MSC aggregation reduced the expansion ratio of ADMSCs during bead-to-bead transfer, whereas the addition of FP003B significantly improved this ratio (Figures 1D,I). Previous studies have reported that increasing the agitation speed from 30 to 60 and 90 rpm decreases the percentage of aggregates, with the highest growth rate observed at 60 rpm (Takahashi et al., 2017). However, higher stirring speeds also increase shear stress, which can negatively affect MSC viability and proliferation and may even cause cell detachment from the MC (Maul et al., 2011). These findings suggest that although increasing agitation speed can reduce MC aggregation, it introduces substantial shear stress that compromises MSC quality. Therefore, physical strategies such as modifying the agitation speed must be applied cautiously to avoid detrimental effects. In contrast, our approach using FP003B suppresses aggregation without increasing mechanical stress, thereby preserving cell integrity and expansion efficiency under serum-free culture conditions. Moreover, a combination of physical and additive strategies could further expand the range of feasible operational parameters. From this perspective, these findings are critical for developing robust and scalable MSC manufacturing processes.

Although FP003B suppressed excessive MC/MSC aggregation when applied through medium exchange (Figures 1, 2–4), this approach increased the number of operational steps (Figure 4A). Process simplification and automation have been reported as essential components of MSC manufacturing (Strecanska et al., 2025). Therefore, we prepared a buffer containing a high concentration of FP003B in advance and investigated whether adding a small volume of this buffer could achieve the same effect as a full medium exchange. The effects of FP003B were comparable between the two methods. These findings may be attributable to the ability of FP003B structures formed at high concentrations in D-PBS (+) to retain a well-dispersed state in the culture medium following their addition and subsequent dilution.

Gellan gum–based materials such as FP001 and FP003 have been used for various cell types, including iPSCs and iPSC-derived intestinal organoids (Otsuji et al., 2014; Ogawa et al., 2022). In these studies, stem cell marker expression, the differentiation potential of iPSCs, lineage-specific intestinal cell markers, and the metabolic and transport functions of iPSC-derived intestinal organoids were maintained. Consistent with these findings in other cell types, the surface marker profiles, trilineage differentiation capacity, and secretion of selected paracrine factors of ADMSCs were also preserved in the presence of FP003B in our study (Figures 1F–H). While these assays do not fully replace functional immunomodulatory potency assays, they provide supportive evidence regarding MSC-related paracrine activity. Together, these reports and our results suggest that FP003B does not obviously impair ADMSC function under the conditions tested, although further detailed evaluation of MSC functionality will be necessary, including both immunomodulatory potency assays and *in vivo* assays.

FP003B is a gellan gum-based functional polymer that forms a weak, ion-dependent network structure in aqueous media. In the presence of divalent cations such as Ca^2+^ and Mg^2+^, the polymer adopts a dispersed particulate or network-like state rather than forming a bulk gel at low concentrations. At the working concentration used in this study (0.005%), FP003B did not measurably increase medium viscosity (control medium: 1.06 mPa·s; FP003B-containing medium: 1.09 mPa·s), whereas methylcellulose increased medium viscosity under the same conditions (0.005% methylcellulose-containing medium: 1.26 mPa·s). These results indicate that FP003B modifies the physical microenvironment without substantially altering bulk viscosity, likely reducing cell–cell interactions on MCs by providing a weakly structured suspension. This property distinguishes FP003B from conventional viscosity-enhancing agents such as methylcellulose (Büyükurgancı et al., 2023) and may underlie its ability to suppress excessive aggregation without markedly affecting bulk hydrodynamic conditions. Collectively, these findings suggest that FP003B is particularly suitable for MC-based expansion systems, as it enables improved control of cell aggregation while maintaining handling properties comparable to standard culture media.

Consistent with these physicochemical properties, FP003B was observed both on the surfaces of MC/MSC aggregates and within the culture medium, even under agitation conditions (Figure 5B; Supplementary Videos S3 and S4). These observations suggest that FP003B associates with MC/MSC surfaces and may act as a cushioning layer that reduces direct contact between MC/MSC particles. This physical effect is consistent with a mechanism in which intercellular distances are maintained, thereby limiting direct physical contact between cells and preventing excessive aggregation. While the present findings are consistent with a surface-associated mechanism of action, alternative explanations may also contribute to the observed effects. For example, FP003B may affect aggregate physical dynamics such as fragmentation upon addition. In addition, contributions from medium components cannot be excluded. These possibilities were not directly evaluated in the present study. Therefore, the proposed mechanism should be considered a working hypothesis rather than a definitive explanation.

ADMSCs have been reported to produce several extracellular matrix (ECM) components, including collagen type I, fibronectin, and laminin, with collagen type I being the predominant constituent (Novoseletskaya et al., 2020). Therefore, we examined whether collagen type I deposition changed when excessive MC/MSC aggregation was suppressed. We found that collagen type I deposition decreased with the addition of FP003B (Figure 5C). Previous studies have demonstrated that MSC spheroids produce higher levels of ECM proteins, such as laminin and fibronectin (Lee et al., 2016), and promote cell–ECM interactions (Ohori-Morita et al., 2025). When excessive aggregation occurs, the proportion of cell–cell and cell–ECM interactions is expected to increase. Based on these reports and our results, we hypothesize that FP003B suppresses excessive aggregation, thereby reducing cell–cell and cell–ECM interactions. This reduction could lead to decreased collagen type I deposition on aggregates and may contribute to enhancing cell recovery. Nevertheless, the quantification of collagen type I based on pepsin digestion may be influenced by differences in aggregate structure. Larger and more compact aggregates, as observed under FP003B (−) conditions, may exhibit increased collagen cross-linking, potentially reducing digestion efficiency and leading to underestimation of collagen content. As a result, the observed differences between conditions may be partially influenced by methodological limitations, and these findings should be interpreted with caution.

In addition to its primary effect on aggregation behavior, FP003B may also influence the cellular microenvironment through physicochemical interactions. As an anionic polymer containing carboxyl groups, FP003B could potentially interact electrostatically with heparin-binding growth factors such as FGF-2, HGF, and EGF, which are known to regulate MSC proliferation and function. Such interactions may affect the local availability, stability, or presentation of these growth factors. Consistent with this possibility, gellan gum-derived polymers have been reported to stabilize heparin-binding growth factors, including FGF-2, through electrostatic interactions (Paluck et al., 2016). However, in the present study, no clear enhancement in cell proliferation was observed based on glucose consumption, lactate production, and NH_4_^+^ accumulation profiles (Figures 1E, 2D). These observations suggest that growth factor-mediated effects alone may not fully explain the observed outcomes. However, potential interactions between FP003B and growth factors were not directly evaluated in the present study and therefore remain speculative.

MSCs have been investigated for treating a wide range of diseases. Intravenous administration is the most employed delivery route in clinical studies (Jovic et al., 2022). The residual amount of process-related additives is an important consideration for cell manufacturing. Preliminary internal observations suggested that residual FP003B levels in harvested ADMSCs may be low under the tested conditions. However, these data were not systematically evaluated and are not included in the present study. In addition, preliminary safety-related assessments, including antigenicity and genotoxicity, have been conducted for FP003B in separate studies, but these data are not included within the scope of the present report. Therefore, while these observations suggest that FP003B may be compatible with cell-manufacturing processes, further detailed safety evaluation will be required.

### 4.1 Limitations

This study represents a pre-clinical proof-of-concept evaluation of FP003B as an aggregation control strategy for MC-based MSC culture. Several limitations should be acknowledged. First, the GMP-grade status of FP003B and its regulatory qualification were not addressed in the present study. Second, although FP003B showed consistent effects across multiple vessel formats, the maximum working volume evaluated (300 mL) remains substantially smaller than clinically relevant manufacturing scales, and further validation in larger-scale bioreactor systems will be required. Third, all experiments in this study were conducted using ADMSCs derived from a single donor, and donor-to-donor variability was not assessed. This may influence MSC behavior and limit the generalizability of the findings. Fourth, although trilineage differentiation and paracrine factor secretion were evaluated, direct functional assays assessing the immunomodulatory potency of ADMSCs (e.g., T Cell suppression assays) were not performed. Therefore, the preservation of immunomodulatory function under FP003B conditions remains to be fully validated. Finally, due to the limited sample size (n = 3–5), assumptions of normality and homogeneity of variance were not formally evaluated, and statistical power to reliably detect moderate effect sizes is limited. In addition, effect size estimates derived from such small samples may be unstable and potentially inflated, which further limits the robustness of the observed differences and warrants cautious interpretation.

## 5 Conclusion

FP003B effectively suppressed excessive MC/MSC aggregation, and it improved cell recovery and expansion efficiency in the serum-free medium. Its applicability across different scales and culture systems highlights its potential for robust, large-scale MSC manufacturing. This approach may become an important technology for pre-clinical proof-of-concept MSC production.

## Data Availability

The original contributions presented in the study are included in the article/Supplementary Material, further inquiries can be directed to the corresponding author.
