# Correction: FP003B suppresses microcarrier aggregation and enhances cell yield in microcarrier-based mesenchymal stromal cell culture

**DOI:** 10.3389/fbioe.2026.2010047

**Published:** 2026-10-08

**Authors:** Frontiers Production Office

**Affiliations:** Frontiers Media SA, Lausanne, Switzerland

**Keywords:** aggregation suppression, FCeM, FP003B, mesenchymal stromal cells, microcarrier

The affiliation of reviewer Andrew Haskell was included incorrectly as “Biomedical Advanced Research and Development Authority (BARDA)”. The correct affiliation is “HAS Consulting, LLC, United States”.

The original version of this article has been updated.

