# Correction: FP003B suppresses microcarrier aggregation and enhances cell yield in microcarrier-based mesenchymal stromal cell culture

**DOI:** 10.3389/fbioe.2026.2008800

**Published:** 2026-10-16

**Authors:** Katsuhiko Kida, Daisuke Hatanaka, Shiho Anno, Miki Nakajima, Masaki Kobayashi

**Affiliations:** 1 Biological Research Laboratories, Nissan Chemical Corporation, Shiraoka, Saitama, Japan; 2 Drug Metabolism and Toxicology, Faculty of Pharmaceutical Sciences, Kanazawa University, Kanazawa, Japan; 3 Chemical Research Laboratories, Nissan Chemical Corporation, Funabashi, Chiba, Japan

**Keywords:** aggregation suppression, FCeM, FP003B, mesenchymal stromal cells, microcarrier

The figure captions were in the wrong order in the PDF and online versions of this paper. The figures themselves were correct, but the caption assigned to Figure 3 belongs to Figure 4, and the caption assigned to Figure 4 belongs to Figure 3. The order has now been corrected.

**FIGURE 3:**
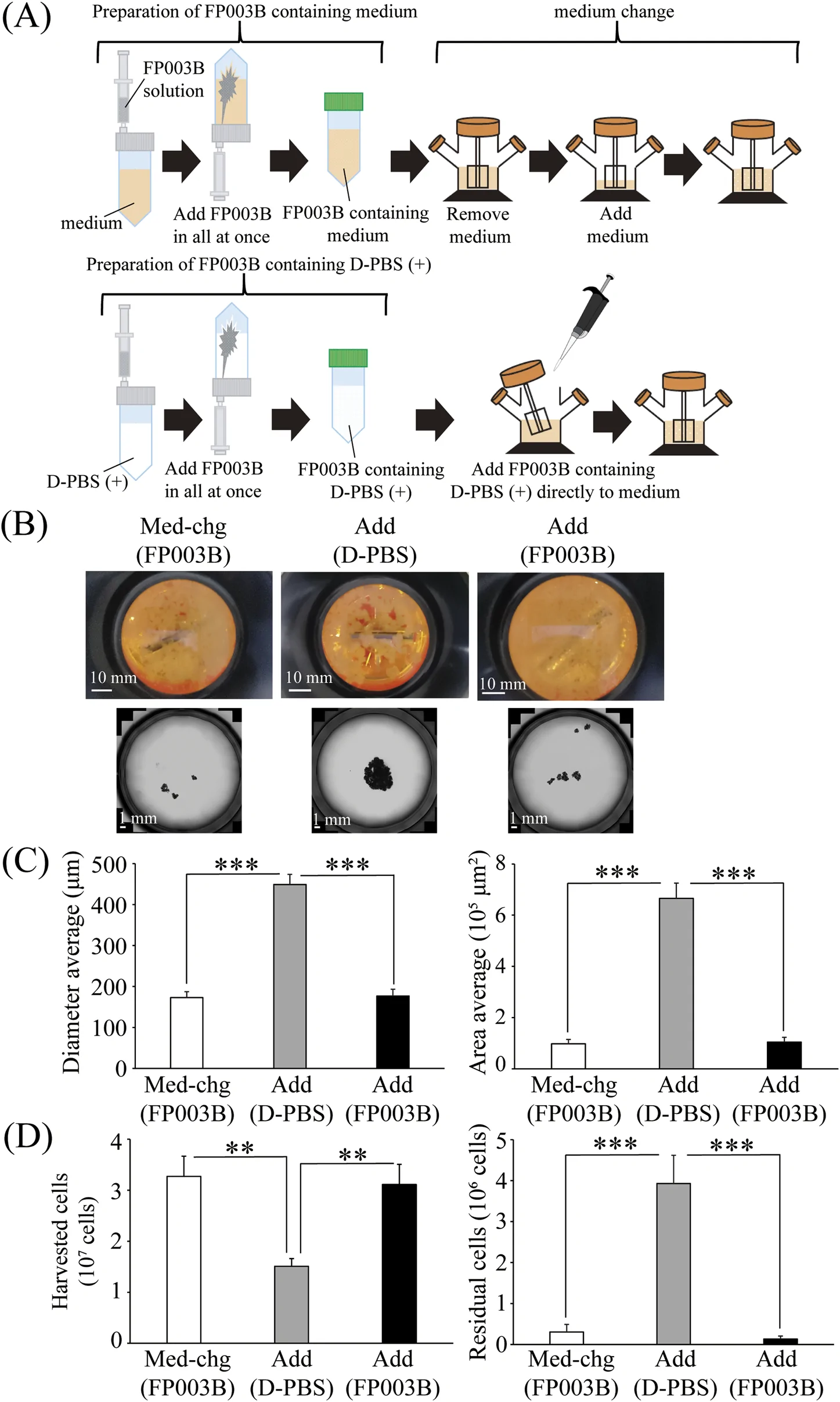
Comparison of methods for FP003B supplementation. **(A)** Schematic illustration of medium change and direct addition methods. The upper panel shows the method of medium change, and the lower panel shows the addition of FP003B containing D-PBS (+). **(B)** Images of the bottom of the spinner flask and MC/MSC aggregates. Med-chg (FP003B), add (D-PBS) and add (FP003B) mean medium change with FP003B, addition of D-PBS (+) only and addition of FP003B containing D-PBS (+), respectively. Scale bars = 10 mm and 1 mm, respectively. **(C)** The average diameter and area of MC/MSC aggregates. ***p < 0.001, compared with add (D-PBS). Bars indicate mean ± SD (n = 3). **(D)** The number of harvested and residual ADMSCs. **p < 0.01, ***p < 0.001, compared with add (D-PBS). Bars indicate mean ± SD (n = 3).

**FIGURE 4:**
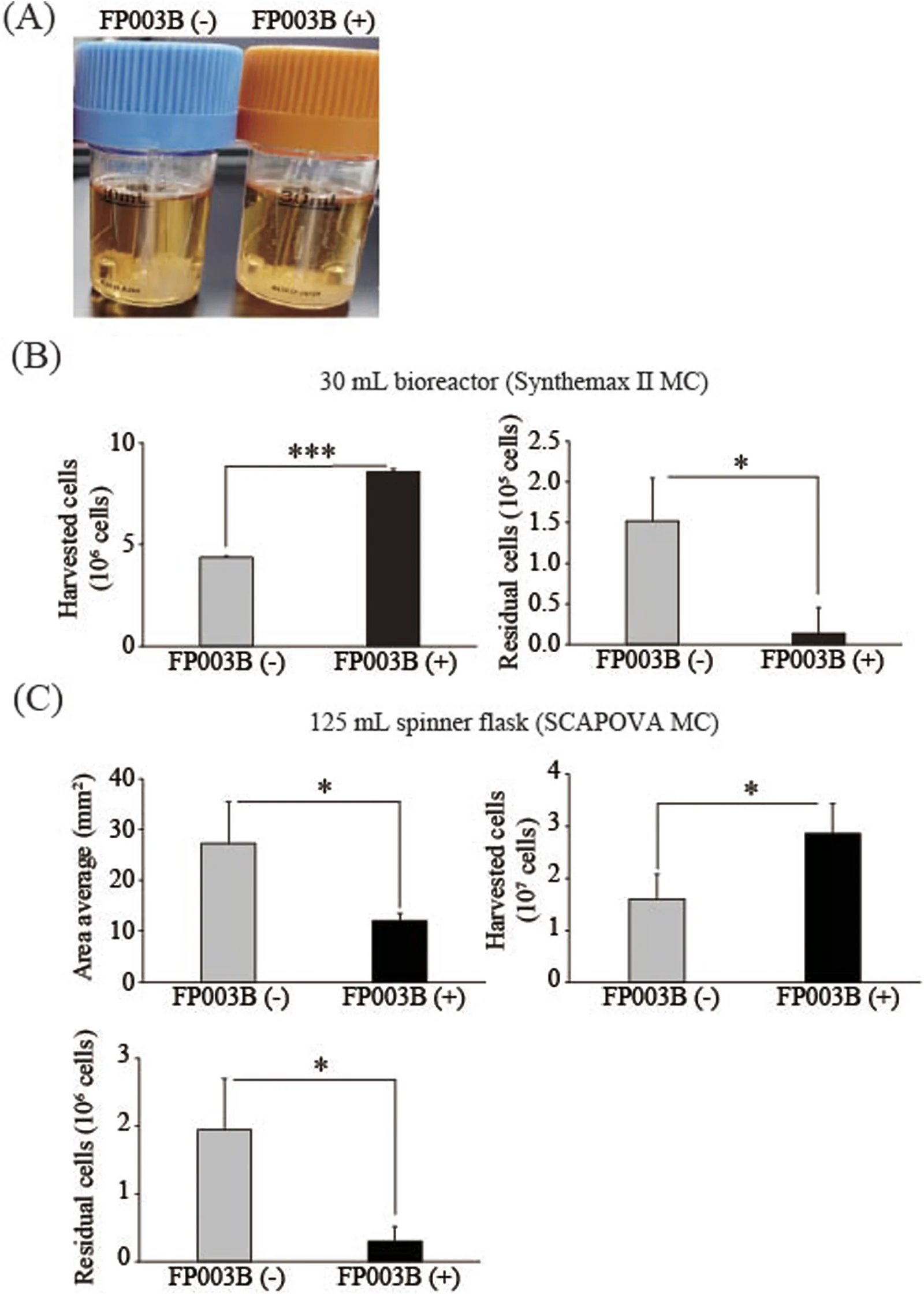
Suppression of MC/MSC aggregation across various types of MCs and culture vessels. **(A)** Images of MC/MSC aggregates in an ABLE 30 mL disposable bioreactor. Left vessel: FP003B (−), Right Vessel: FP003B (+). **(B)** The number of harvested and residual ADMSCs using an ABLE 30 mL disposable bioreactor. *p < 0.05, ***p < 0.001, compared with FP003B (−). Bars indicate mean ± SD (n = 3). **(C)** The average diameter of MC/MSC aggregates and the number of harvested and residual ADMSCs using SCAPOVA MC. *p < 0.05, compared with FP003B (−). Bars indicate mean ± SD (n = 3).

In addition, the caption for Figure 4 incorrectly separated panel C into panels C and D. The correct caption is:

“(C) The average diameter of MC/MSC aggregates and the number of harvested and residual ADMSCs using SCAPOVA MC. *p < 0.05, compared with FP003B (−). Bars indicate mean ± SD (n = 3)”.

The original version of this article has been updated.

