# Supplementary material for: FP003B suppresses microcarrier aggregation and enhances cell yield in microcarrier-based mesenchymal stromal cell culture
Source: Front Bioeng Biotechnol. 2026 Sep 3;14:1881013. doi: 10.3389/fbioe.2026.1881013 (PMC13582311; doi:10.3389/fbioe.2026.1881013)
Supplement: Supplementary file 1 [file Supplementaryfile1.docx]

Supplementary Material

## Supplementary Videos

**Supplementary Video 1.** A video of a 125 mL flask during agitation culture in FP003B (-) condition.

**Supplementary Video 2.** A video of a 125 mL flask during agitation culture in FP003B (+) condition.

**Supplementary Video 3.** A video of FP003B in the culture medium during agitation culture being analyzed by a PIV laser

**Supplementary Video 4.** An overlaid image of FP003B localization on MC and ADMSC surfaces. Green indicates FITC-labeled FP003B, and blue indicates stained live ADMSCs.

## Supplementary Figures

**Supplementary Figure 1.** Representative staining images at the initiation of differentiation (Day 0): Oil-Red O, Alizarin Red S and Alcian Blue. Scale bar = 200 μm and 500 μm, respectively.

## Supplementary Tables

**Supplementary Table 1.** Statistical summary of quantitative analyses corresponding to Figures 1, 2 and 4. The statistical comparisons of selected endpoints (diameter, area, harvested cells, residual cells, and total cell number) across experimental conditions are presented. For each comparison, raw p-values and adjusted p-values (adj p-value) are reported together with the mean difference and 95% confidence interval (CI). Effect sizes are presented as Cohen’s d calculated based on indicated sample sizes (n = 3 or n = 5, where applicable). For Figures 1C and 1D, experiments were repeated with n = 5 independent biological replicates to strengthen statistical robustness; results obtained with n = 3 are provided for reference and transparency.

**Supplementary Table 2.** Quantitative flow cytometry analysis of MSC surface marker expression. The percentages of cells positive for MSC surface markers (CD73, CD90, and CD105) and negative markers (CD11b, CD34, and CD45) are presented as mean ± SD (n = 3). Data were obtained from independent biological replicates and analyzed under FP003B (-) and FP003B (+) conditions. These data complement the representative histograms shown in Figure 1F. These results support the maintenance of MSC surface marker expression under both conditions.
